# Dermal Denticle Diversity in Sharks: Novel Patterns on the Interbranchial Skin

**DOI:** 10.1093/iob/obab034

**Published:** 2021-12-22

**Authors:** Molly K Gabler-Smith, Dylan K Wainwright, Greta A Wong, George V Lauder

**Affiliations:** Department of Organismic and Evolutionary Biology, Harvard University, 26 Oxford Street, Cambridge, MA, 02138 USA; Department of Ecology and Evolutionary Biology, Yale University, 165 Prospect Street, New Haven, CT, 06511 USA; Department of Organismic and Evolutionary Biology, Harvard University, 26 Oxford Street, Cambridge, MA, 02138 USA; Department of Organismic and Evolutionary Biology, Harvard University, 26 Oxford Street, Cambridge, MA, 02138 USA

## Abstract

Shark skin is covered in dermal denticles—tooth-like structures consisting of enameloid, dentine, and a central pulp cavity. Previous studies have demonstrated differences in denticle morphology both among species and across different body regions within a species, including one report of extreme morphological variation within a 1 cm distance on the skin covering the branchial pouches, a region termed “interbranchial skin.” We used gel-based profilometry, histology, and scanning electron microscopy to quantify differences in denticle morphology and surface topography of interbranchial skin denticles among 13 species of sharks to better understand the surface structure of this region. We show that (1) interbranchial skin denticles differ across shark species, and (2) denticles on the leading edge of the skin covering each gill pouch have different morphology and surface topography compared with denticles on the trailing edge. Across all species studied, there were significant differences in denticle length (*P* = 0.01) and width (*P* = 0.002), with shorter and wider leading edge denticles compared with trailing edge denticles. Surface skew was also higher in leading edge denticles (*P* = 0.009), though most values were still negative, indicating a surface texture more dominated by valleys than peaks. Overall, leading edge denticles were smoother-edged than trailing edge denticles in all of the species studied. These data suggest two hypotheses: (1) smoother-edged leading edge denticles protect the previous gill flap from abrasion during respiration, and (2) ridged denticle morphology at the trailing edge might alter water turbulence exiting branchial pouches after passing over the gills. Future studies will focus on determining the relationship between denticle morphology and water flow by visualizing fluid motion over interbranchial denticles during *in vivo* respiration.

## Abstract (pt)

La piel de los tiburones está cubierta de dentículos dérmicos, estructuras similares a los dientes que constan de un tejido esmaltado, una dentina y una cavidad pulpar central. Estudios anteriores han demostrado diferencias en la morfología de los dentículos tanto entre especies como entre diferentes regiones del cuerpo dentro de una misma especie, incluyendo un informe sobre la extrema variación morfológica dentro de una distancia de 1 cm en la piel que cubre las bolsas branquiales, una región denominada "piel interbranquial." Hemos utilizado perfilometría basada en gel, histología y microscopía electrónica de barrido, para cuantificar las diferencias en la morfología de los dentículos y la topografía de la superficie de la piel interbranquial de los dentículos en 13 especies de tiburones, para comprender mejor la estructura de la superficie de esta región. Demostramos que (1) los dentículos de la piel interbranquial difieren entre las especies de tiburones, y (2) los dentículos del borde anterior de la piel que cubre cada bolsa branquial tienen una morfología y una topografía superficial diferentes en comparación con los dentículos del borde posterior. En todas las especies estudiadas, hubo diferencias significativas en la longitud (*P* = 0.01) y en el ancho (*P* = 0.002), con dentículos del borde anterior más cortos y anchos que los del borde posterior. La inclinación de la superficie también era mayor en los dentículos del borde anterior (*P* = 0.009) aunque la mayoría de los valores seguían siendo negativos, lo que indicaba más valles que picos. En general, los dentículos de la parte anterior tenian los bordes mas lisos que los de la parte posterior en todas las especies estudiadas. Estos datos sugieren dos hipótesis: (1) los dentículos del borde anterior con bordes más lisos protegen la aleta branquial previa de la abrasión durante la respiración, y (2) la morfología de los dentículos con crestas en el borde posterior podría alterar la turbulencia del agua que sale de las bolsas branquiales después de pasar por las branquias. Futuros estudios se centrarán en determinar la relación entre la morfología de los dentículos y el flujo de agua mediante la visualización del movimiento del fluido sobre los dentículos interbranquiales durante la respiración in vivo.

Translated by Laura Paez, Ph.D. student—Swiss Federal Institute of Technology Lausanne

## Abstract (es)

상어의 비늘은 유법랑질, 상아질, 치수강으로 이루어졌으며, 낱개로 보면 생김새가 이빨을 닮았다. 기존의 연구에서는 상어 비늘의 형태학적 구조를 종 간 및 동종 내 다른 어체 부위의 다양한 각도에서 분석했는데, 그 중에서도 아가미구멍 사이의 1cm에 불과한 범위에서 예상 외의 표면미세구조 다양성을 발견한 연구가 주목된다. 상어 비늘의 형태학적 이해를 돕기 위하여, 본 연구에서는 젤을 사용한 프로필로메트리(gel-based profilometry), 조직학적 기법 및 주사전자현미경법을 통하여, 상어류 13종에서 아가미구멍 사이 피부의 비늘 형태와 표면미세구조를 분석했다. 본 연구의 결과는 (1) 아가미구멍 사이 피부의 표면형태에는 종 간 차이가 있고, (2) 아가미구멍 사이 피부의 전방 (머리 방향) 비늘은 후방 (꼬리 방향) 의 비늘에 비하여 훨씬 더 큰 수준의 다양성을 보였다는 것이다. 분석한 13종 모두를 통틀어, 아가미구멍 사이 피부의 전방 비늘은 후방의 비늘 보다 훨씬 넓고 (*P* = 0.01) 길었다 (*P* = 0.002). 본 연구에서는 (1) 부드러운 머리 쪽 비늘이 상어가 숨 쉴 때마다 아가미구멍을 통해 빠져나온 물 흐름의 저항을 줄여준다는 것, 그리고 (2) 꼬리 쪽 가장자리의 비늘에서 두드러지는 들쭉날쭉한 가장자리는 아마도 비슷한 원리에서 아가미구멍을 통해 빠져나온 물의 소용돌이를 줄여 준다는 가설을 세울 수 있었다. 미래의 연구에서는 실험실 내의 조건에서 아가미구멍을 통해 흐르는 물의 역학적인 측면을 상어 비늘의 형태학적 측면과 연관지어 접근해야 할 것이다.

Translated by Daemin Kim, Ph.D. student—Yale University

Die Haut von Haien ist mit dermalen Dentikeln bedeckt - zahnähnlichen Strukturen, die aus Schmelz, Dentin und einer zentralen Pulpahöhle bestehen. Vorhergehende Studien haben Unterschiede in der Morphologie der Dentikel sowohl zwischen den Arten als auch zwischen verschiedenen Körperregionen innerhalb einer Art gezeigt, einschließlich eines Berichts über extreme morphologische Variationen innerhalb eines Abstands von 1 cm auf der Haut, die die Kiementaschen bedeckt, eine Region, die als "Interbranchialhaut" bezeichnet wird. Um die Oberflächenstruktur dieser Region besser zu versteshen, haben wir die Unterschiede in der Morphologie und Oberflächentopographie der Dentikel der Interbranchialhaut in 13 Haiarten mit Hilfe von Gel-Profilometrie, Histologie und Rasterelektronenmikroskopie quantifiziert. Wir konnten zeigen, dass (1) sich die Dentikel der Interbranchialhaut zwischen den Haiarten unterscheiden und (2) die Dentikel an der Vorderkante der Haut, die jede Kiementasche bedeckt, eine andere Morphologie und Oberflächentopographie aufweisen als die Dentikel an der Hinterkante. Bei allen untersuchten Arten gab es signifikante Unterschiede in der Länge (*P* = 0.01) und Breite (*P* = 0.002) der Dentikel, wobei die Dentikel an der Vorderkante kürzer und breiter waren als die Dentikel an der Hinterkante. Auch die Oberflächenschiefe war bei den Dentikeln der Vorderkante höher (*P* = 0.009), obwohl die meisten Werte immer noch negativ waren, was auf mehr Täler als Spitzen hinweist. Insgesamt waren die Vorderkanten-Dentikel bei allen untersuchten Arten glatter als die Hinterkanten-Dentikel. Diese Daten legen zwei Hypothesen nahe: (1) Glattere Vorderkantenzähne schützen den vorhergehenden Kiemenlappen vor Abrieb während der Atmung, und (2) die Morphologie der gezackten Zähne an der Hinterkante könnte die Wasserturbulenz beim Austritt aus den Kiementaschen nach dem Passieren der Kiemen verändern. Zukünftige Studien werden sich darauf konzentrieren, die Beziehung zwischen der Morphologie der Dentikel und der Wasserströmung zu bestimmen, indem die Flüssigkeitsbewegung über die Interbranchialdentikel während der In-vivo-Atmung sichtbar gemacht wird.

Translated by Robin Thandiackal, postdoctoral fellow—Harvard University

La peau des requins est recouverte de denticules dermiques - des structures semblables à des dents composées d'émail, de dentine et d'une cavité pulpaire centrale. Des études précédentes ont démontré que la morphologie des denticules diffère entre les espèces, mais également entre les différentes régions du corps au sein d'une même espèce. Il existe notamment une variation morphologique extrême sur une distance de 1 cm dans la région appelée "peau interbranchiale," soit la peau peau couvrant les poches branchiales. Nous avons utilisé la profilométrie à base de gel, l'histologie et la microscopie électronique à balayage pour quantifier les différences morphologiques et topographiques des denticules de la peau interbranchiale chez 13 espèces de requins, ceci afin de mieux comprendre la structure de la surface de cette région. Nos résultats montrent que (1) les denticules de la peau interbranchiale diffèrent selon les espèces de requins, et (2) les denticules situées sur le bord d'attaque de la peau couvrant chaque poche branchiale ont une morphologie et une topographie de surface différentes de celles des denticules situées sur le bord de fuite. Chez toutes les espèces étudiées, il y avait des différences significatives dans la longueur (*P* = 0.01) et la largeur (*P* = 0.002) des denticules, avec les denticules du bord antérieur plus courtes et plus larges que celles du bord postérieur. L'asymétrie de la surface était également plus élevée dans les denticules antérieures (*P* = 0.009), bien que la plupart des valeurs soient négatives, indiquant plus de vallées que de sommets.Par ailleurs, , les denticules du bord antérieur étaient plus lisses que celles du bord postérieur. Dans l'ensemble, ces données suggèrent deux hypothèses: (1) les denticules situées sur le bord d'attaque et possédant une surface plus lisse protègent le volet branchial précédent de l'abrasion pendant la respiration, et (2) la morphologie plutôt striée des denticules situées sur le bord de fuite pourrait modifier les caractéristiques turbulentes de l'écoulement sortant des poches branchiales après être passé sur les branchies. Les études futures se concentreront sur la détermination de la relation entre la morphologie des denticules et l’écoulement de l'eau en visualisant le mouvement du fluide sur les denticules interbranchiaux pendant la respiration in vivo.

Translated by Elsa Goerig, postdoctoral fellow—Harvard University

## Introduction

One of the most remarkable aspects of shark biology is the structure of their unique skin. The skin of sharks is covered in many thousands of dermal denticles, which are tooth-like structures composed of enameloid and dentine outer layers and a central pulp cavity. Denticles have a characteristic form, which consists of a flattened crown and an elongated neck that extends to an expanded base embedded into the dermis. Though all shark denticles have a generally similar structure, there is considerable diversity in denticle shape, size, and density within and among shark species (Ankhelyi et al. 2018; Bigelow and Schroeder 1948; Díez et al. 2015; Lang et al. 2012; Motta et al. 2012; Oeffner and Lauder 2012; Raschi and Tabit 1992; Reif 1985a,b).

Along with studies on the diversity of denticle morphology, there have been many proposed functions of dermal denticles, including abrasion reduction, protection from predators and ectoparasites, and use of denticles during feeding and mating (Pratt and Carrier 2001; Raschi and Tabit 1992; Southall and Sims 2003; Tricas and Le Feuvre 1985). One function that has been an important focus for research is the role of denticles in support of locomotion, as many extant shark species have denticles with morphologies that improve swimming performance. Fluid dynamic studies have revealed that denticles can improve swimming performance by enhancing thrust, reducing hydrodynamic drag, as well as changing the boundary layer characteristics of water flow over the body (DuClos et al. 2018; Lang et al. 2012; Lauder et al. 2016; Oeffner and Lauder 2012). Hydrodynamic studies using foils covered in real pieces of shark skin or three-dimensional (3D)-printed shark skin models have offered an additional understanding of how denticles may function in flow (Domel et al. 2018; Lauder et al. 2016; Oeffner and Lauder 2012; Wen et al. 2014, 2015). However, denticles are morphologically diverse and there is still uncertainty as to how changes in denticle morphology may affect function and performance. In part, this lack of knowledge remains due to the challenging nature of both understanding patterns of the 3D morphology of shark surfaces and experimentally examining flows over denticle surfaces—body deformation during swimming coupled with the need for a very small field of view to observe flow over small patches of denticles makes visualizing natural flows difficult.

Although connecting denticle diversity with *in vivo* flows in sharks is challenging, a recently published image of denticles on a segment of skin between gill slits (the “interbranchial” or “branchial” skin) provides hope in that regard. In that report, a single image taken from the interbranchial skin of a smooth dogfish (*Mustelus canis*; Ankhelyi et al. 2018) shows a dramatic gradient in denticle shape. Specifically, rounded, smooth-edged denticles were found along the leading edge of the branchial skin, and more triangular denticles with surface ridges were found along the trailing edge, all over a distance of just a few millimeters. Interestingly, the diversity observed in only a few millimeters at the interbranchial skin seems to replicate the diversity of denticle forms that have been observed around the entire body in other species. For example, in species like the smooth dogfish and thresher shark, denticles from the leading edge of the tail and fins tend to be flattened, more rounded, and have reduced ridges compared with the denticles on the trailing edges, which are triangular with multiple ridges (Ankhelyi et al. 2018; Popp et al. 2020; Reif 1985b). If denticle diversity is similar on the interbranchial skin of other individuals and species, the interbranchial region may be an interesting target for simultaneously imaging surface flows above denticles of different morphology. Moreover, substantiating the discovery of denticle diversity at the interbranchial region in sharks would add to our growing knowledge about comparative patterns of denticle diversity across shark bodies and species. Unfortunately, current data on the interbranchial skin are very limited—several studies describe the general morphology of internal denticles within the mouth cavity and on gill rakers (Nelson 1970; Paig-Tran and Summers 2014), but just the single aforementioned study (Ankhelyi et al. 2018) shows data for the interbranchial skin, and just from a single image of one species.

Flow over the interbranchial skin would likely be dominated by respiratory flows. During respiration, high volumes of water are taken in through the mouth and are expelled through the gill slits while the interbranchial skin located between adjacent gill slits undergoes considerable deformation as a result of the regular expansion and compression of the branchial chambers by constrictor muscles (Liem and Summers 1999). Flow exiting the gills then passes over denticles located on the interbranchial region between gill slits. The pumping of water across the gills is a common behavior for most sharks and is called active ventilation. This is contrasted with ram ventilation, where a shark swims forward with enough speed to pass flow through the mouth and across the gills (Graham et al. 1990; Wegner et al. 2012). Some shark species are obligate ram ventilators, but most are active ventilators, especially at zero or low swimming speeds (Barker et al. 2011; Ferry-Graham 1999; Roberts 1975; Tomita et al. 2018; Wegner et al. 2012). Because even stationary sharks experience regular respiratory flows, the interbranchial skin may be a tractable system for studying fluid flow in live sharks due to the relative ease of visualizing boundary layer and near-skin flows when the shark is not swimming. Future studies may then find it possible to experimentally measure flow in the interbranchial skin region and correlate patterns with denticle morphology, particularly in sedentary, benthic species.

Therefore, there are two main goals of this study. First, we imaged and quantified surface topography of interbranchial skin denticles across 13 different shark species to determine whether the gradient in denticle morphology observed previously occurs in a diversity of shark species. These shark species also exhibit a range of ecologies (e.g., benthic, demersal, pelagic, suspension feeding), respiratory modes (e.g., active and ram ventilation), and locomotor modes (e.g., sedentary and active; Barker et al. 2011; Dolce and Wilga 2013; Graham et al. 1990; Roberts 1975; Thomson and Simanek 1977; Tomita et al. 2018; Wegner et al. 2012). In doing so, we describe denticle morphology and provide quantitative measurements of surface topography from branchial skin denticles from multiple species at multiple locations around the gills and body. Second, we investigated potential differences in denticle morphology and surface topography between the leading edge and trailing edge denticles on the interbranchial skin in an effort to elucidate morphological patterns across species and the possible functional roles for differences in denticle morphology on interbranchial skin. Perhaps differences in surface topography (rough versus smooth surfaces) between the leading and trailing edges may influence fluid dynamic drag. Since the branchial region experiences routine oscillatory flow (Ferry-Graham 1999), any change in denticle morphology and surface characteristics could suggest functional differences between leading and trailing edge locations and generate testable ideas for future experimental work.

## Materials and methods

### Study animals

**Fig. 1:**
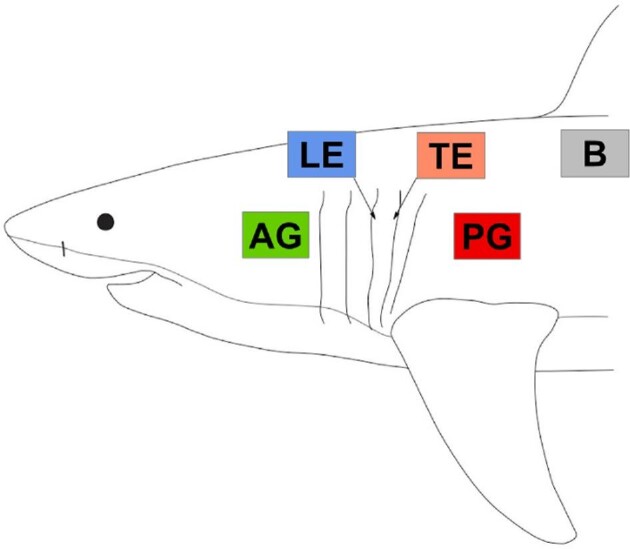
Regions of the body and gills where samples were collected for denticle surface profilometry and size measurements. Outline of a white shark (*Carcharodon carcharias*) indicating where the five regions of tissue were sampled. AG = anterior to gill slit 1 (in green), LE = leading edge of interbranchial skin between gill slits 3 and 4 (in blue), TE = trailing edge of interbranchial skin between gill slits 3 and 4 (in orange), PG = posterior to gill slit 5 (in red), and B = body under the first dorsal fin (in gray).

Data for this study were obtained from fish caught from fishing surveys (under National Marine Fisheries Scientific Permit #HMS-SRP-18-04), biodiversity surveys by the US National Marine Fisheries Service (NOAA), and specimens that were frozen or stored in 70% ethanol from the Harvard Museum of Comparative Zoology (MCZ) Ichthyology Collection. All frozen specimens were placed in sealed bags to prevent desiccation. The interbranchial skin on the head (located between gill openings) and skin from several specific locations on the body (Fig. 1) from 13 species of sharks was analyzed (see Fig. 1 and Table S1 for details about sampling). These species included: one basking shark (*Cetorhinus maximus*, stored in 70% ethanol, MCZ Ichthyology #54413), one bonnethead shark (*Sphyrna tiburo*, frozen), three chain catsharks (*Scyliorhinus retifer*, frozen), one juvenile leopard shark (*Triakis semifasciata*, stored in 70% ethanol), three porbeagles (*Lamna nasus*, frozen), one sand tiger (*Carcharhinus taurus*, frozen), two shortfin mako sharks (*Isurus oxyrinchus*, frozen), one silky shark (*Carcharhinusfalciformis*, stored in 70% ethanol, MCZ Ichthyology #40787), one small-spotted catshark (*Scyliorhinus canicula*, stored in 70% ethanol, MCZ Ichthyology #57053), three smooth dogfish (*Mustelus canis*, frozen), one spiny dogfish (*Squalus acanthias*, preserved and subsequently frozen), one thresher shark (*Alopias vulpinus*, frozen), and one white shark (*Carcharodon carcharias*, stored in 70% ethanol, MCZ Ichthyology #171013). Unless otherwise noted, all individuals were subadults or adults.

We also categorized the general habitat and ecology of our sampled species to see whether there are ecologically related patterns in interbranchial skin denticle morphology. We defined four broad categories: benthic, demersal, pelagic, and suspension. Additionally, since the current study is focusing on the interbranchial skin where respiration occurs, we also wanted to consider each group's ventilatory behavior along the axis from active suction ventilation to ram ventilation (see Dolce and Wilga 2013). Benthic species spend much of their time resting on the benthos and likely only use active ventilation (small-spotted catshark and chain catshark). Demersal species include species that sometimes rest on the benthos, but are most often swimming or station-holding, typically near a structure (sand tiger, smooth dogfish, bonnethead, spiny dogfish, and leopard shark). Demersal species are typically active ventilators that can switch to ram ventilation at higher swimming speeds. Pelagic species generally swim in open water, are capable of high-performance swimming, and are likely obligate ram ventilators (common thresher, white shark, silky shark, shortfin mako, and porbeagle). Suspension feeders are large-bodied, slow-swimming sharks (basking shark) and are likely ram ventilators. We placed suspension feeders in their own category due to their modified gill anatomy as a result of their feeding strategy, which entails swimming slowly and passing a large amount of water over the gills (Paig-Tran and Summers 2014).

### Surface profilometry

Our goal was to explore differences in denticle morphology and surface texture on the interbranchial skin and surrounding body skin among shark species and assess the extent to which denticles on interbranchial skin vary in morphology. To meet this goal, we collected data from five general regions near the gill openings: the interbranchial skin between gill openings (either between gill openings 2–3 or 3–4) including leading edge (LE) and trailing edge (TE) regions, the region anterior to the first gill opening (AG), the region posterior to the last gill opening (PG), and the body (B) region on the lateral side of the body ventral to the first dorsal fin (Fig. 1). We sampled 13 species across a diversity of shark clades (see the “Study animals” section earlier) but in some cases were unable to sample all five selected regions on an individual due to incomplete specimens (see Table S1 for details). Samples used for surface profilometry were obtained either by removing sections of skin approximately 4 cm × 4 cm in size or by collecting data with the skin *in situ* on the specimen. We used gel-based profilometry to image these five regions in order to collect data on denticle morphology and surface texture.

Gel-based profilometry involves pressing a deformable clear elastomer gel with a painted bottom surface onto a region of interest (GelSight Incorporated, Waltham, MA). The painted side of the gel conforms to the surface and then photographs are taken at six different illumination angles. Images are then processed with GelSight software into 3D, topographic surfaces. Following previous methodology (Ankhelyi et al. 2018; Popp et al. 2020; Wainwright et al. 2017, 2019; Wainwright and Lauder 2017), surface metrology variables were quantified and 3D skin surface topography was described.

After we acquired the topographic images, 3D surfaces were processed using MountainsMap (v. 7 Digital Surf, Besançon, France). Within each image, three spatially separate 800 μm^2^ areas were cropped and analyzed, providing nested measurements for each topographic image (see the “Statistical analyses” section later). Large-scale background curvature was removed from the surfaces, and we measured several metrology variables on each cropped image, including root-mean-square roughness (Sq), skew (Ssk), and kurtosis (Sku). We also quantified denticle morphology by measuring average length, average width, and aspect ratio (length/width) of five denticles for each of the five regions (Fig. 1) using ImageJ (NIH, Bethesda, MD). Ridge spacing and height were not measured in these samples as data for many of the species analyzed in the current paper have had denticle ridge spacing and height previously documented (see Ankhelyi et al. 2018; Domel et al. 2018; Popp et al. 2020).

The surface metrology variables we used are standard parameters to report when describing surfaces and we describe them briefly here (see also ISO 25178-2 2012). Root-mean-square roughness values are calculated by taking the squared distance of each point from the mean height and then calculating the square root of the sum across that surface (measured in *µ*m). Skew and kurtosis are unitless variables that describe the distribution of heights across the surface. For example, surfaces with a normal distribution of heights will have a skew of zero and a kurtosis of three. Surfaces with positive skew values have textures that are dominated by peaks (positive surface features) while surfaces with negative skew values have textures that are dominated by valleys (negative surface features). Kurtosis values greater than three indicate surfaces with very high peaks and very low valleys, while values less than three indicate surfaces with less extreme variation than expected under a normal distribution of heights (Dotson 2015; Raghavendra and Krishnamurthy 2013; Westfall 2014).

### Histology and microscopy

Skin samples were taken from the third interbranchial skin region (between gill slits 3 and 4) from two *S. retifer* individuals. These samples were fixed in paraformaldehyde and embedded in paraffin. Ten μm thick sections were stained with hematoxylin and eosin or Masson's trichrome stain. Images of the prepared slides were taken using a Nikon D7000 attached to a Leica DM 2500 P compound microscope (Leica Microsystems, Wetzlar, Germany) at either ×200 or ×400 magnification. For scanning electron microscopy, small samples were removed from each of the interbranchial regions in one individual of *S. retifer*, critical point dried, sputter coated, and imaged at magnifications ranging from ×100 to ×700. The chain catshark shows a particularly dramatic transition in denticle morphology across the interbranchial region, so we focused our histological analysis on this one species, although we would expect similar histological results in other shark species since interbranchial denticles exhibit all major features of body skin surface denticles.

### Statistical analyses

Nested analyses of variance (ANOVAs) with individual as a nested random effect were used to determine whether the five regions studied show differences in denticle length, aspect ratio (length/width), or root-mean-square roughness in either smooth dogfish (*n* = 3 individuals) or porbeagle (*n* = 3 individuals) specimens. In addition, we pooled LE and TE data across all species and used nested ANOVAs with species and individual as nested random effects to determine whether LE and TE measurements are different across the 13 species of sharks. We also pooled LE and TE data into groups according to our ecological categories (benthic, demersal, pelagic, and suspension feeding) and again used nested ANOVAs with individual and species as nested random effects to determine whether ecological groups have any effect on denticle morphology between the LE and TE. For all comparisons, where applicable, post-hoc tests were used to determine differences between groups. All analyses were conducted using the statistical software R (ver. 4.0.1, “See Things Now”; R Foundation for Statistical Computing, Vienna, Austria). Nested ANOVAs and post-hoc tests (least square means pairwise comparisons with a Tukey correction on *P*-values) were implemented using the “nlme,” “lsmeans,” and “multcomp” packages in R (Hothorn et al. 2008; Lenth 2016; Pinheiro et al. 2020; R Core Team 2020). Test values were considered significant at *P* ≤ 0.05.

## Results

**Fig. 2:**
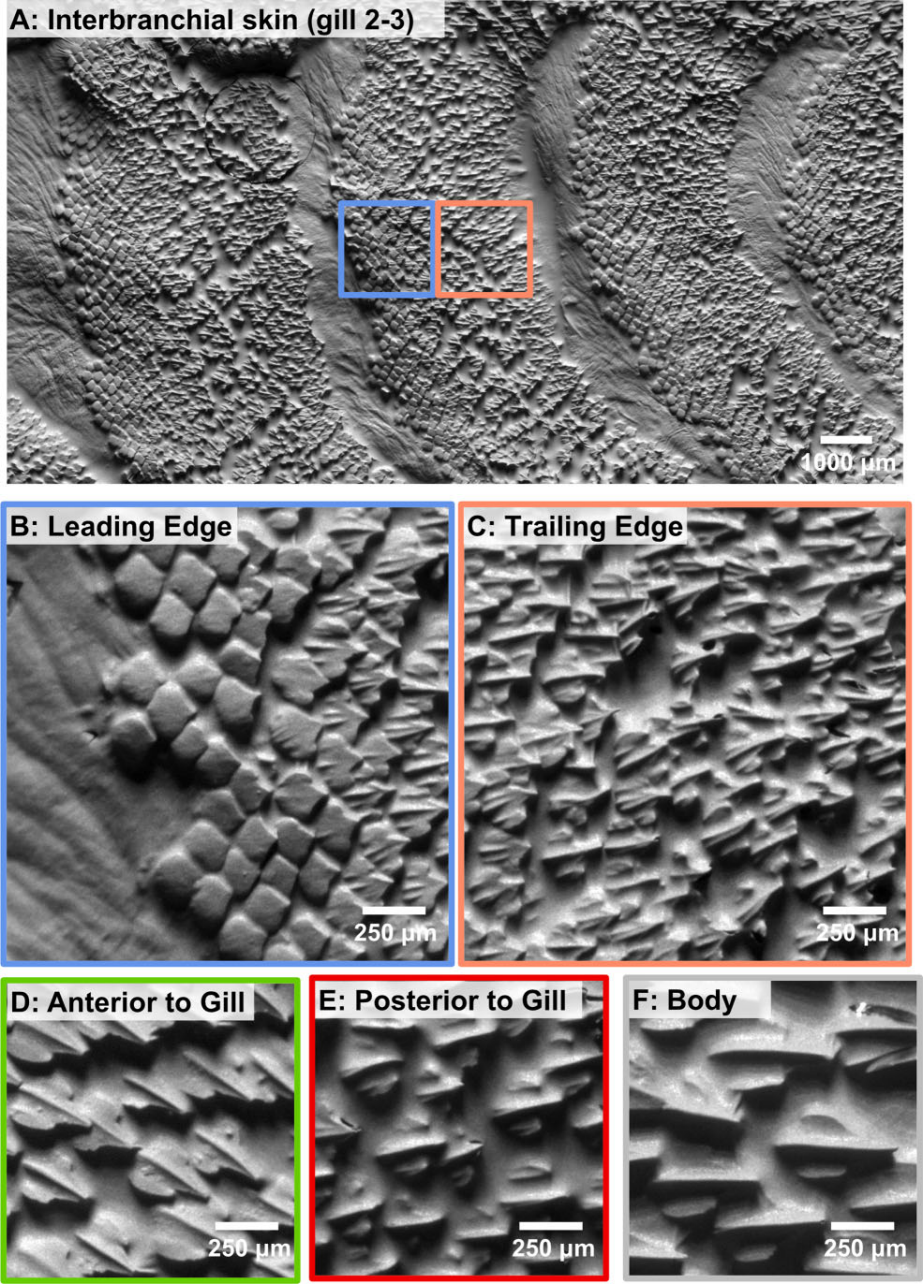
Images of denticles (taken by surface profilometry) from the five regions on the leopard shark (*Triakis semifasciata*). Panel **(A)** shows the entire interbranchial skin surface imaged between gill slits 2 and 3. The colored boxes are magnified in panels **(B)** and **(C)** to illustrate the denticles in the leading and trailing edge interbranchial skin regions. Panels **(B–F)** show the denticle surface corresponding to the sampling locations from Fig. 1.

First, we present general morphological data on denticles from the interbranchial and surrounding regions in the leopard shark. These data will demonstrate the variation in denticle surface topography within an individual at the five regions sampled in this study. The leopard shark was chosen because the specimen is small enough to allow the entire interbranchial skin surface to be imaged in one Figure (Fig. 2A). We then begin each subsection with results from one individual species before broadening the analysis to include data from multiple shark species.

### Morphology and surface characteristics of denticles around the gills

**Fig. 3:**
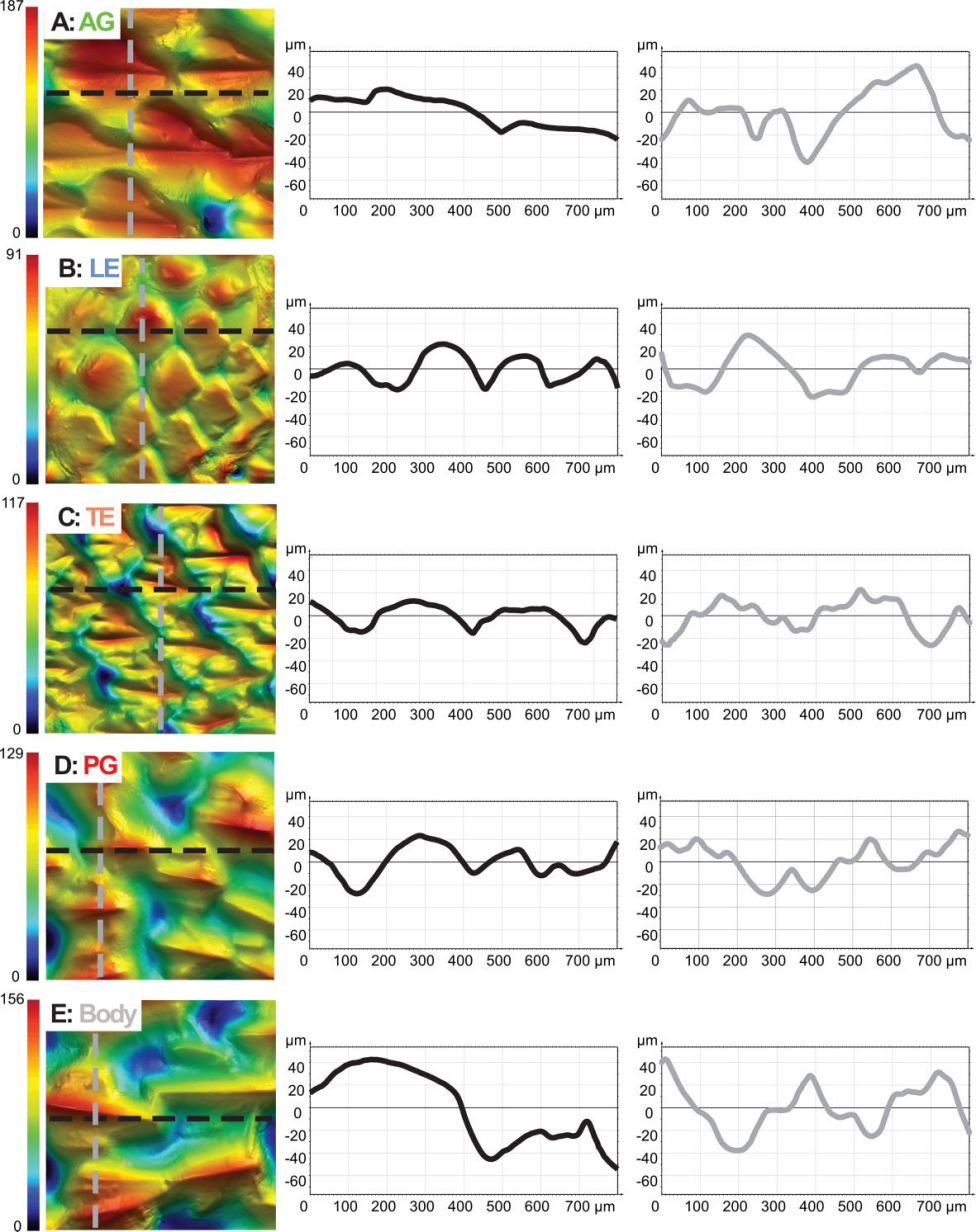
Surface topography and height profiles from the five sampled regions of a leopard shark (*Triakis semifasciata*), corresponding to panels (B–F) in Fig. 2. The left column shows surface topography with the height colored and scale bars with minimum and maximum height values (in μm). Anterior is to the left and dorsal is at the top in all images. All images have an area of 800 μm^2^. The middle and right columns show height profiles for each surface at the two positions indicated by the black (horizontal) and gray (vertical) lines. For these profile lines, zero height is the mean surface height.

Surface images from the five regions of interest in a leopard shark are shown in Fig. 2 with their corresponding height maps and surface profiles shown in Fig. 3. These images illustrate differences in denticle shape, size, and surface topography between the leading (LE) and trailing edges (TE) of the interbranchial skin surface as well as differences in other skin regions (AG = anterior to gill slit 1, PG = posterior to gill slit 5, and B = body (Fig. 2B–F). A gradient in denticle morphology is clearly seen across the entire surface of the branchial gill skin (Fig. 2A). Variation in denticle surface ornamentation, size, and shape are evident across all regions (Figs. 2B–F and 3). Qualitatively, leopard shark LE denticles are smooth-edged and spatulate, and lack any type of ridge (Figs. 2B and 3B). In contrast, TE denticles are more triangular, with multiple ridges (Figs. 2C and 3C).

**Fig. 4:**
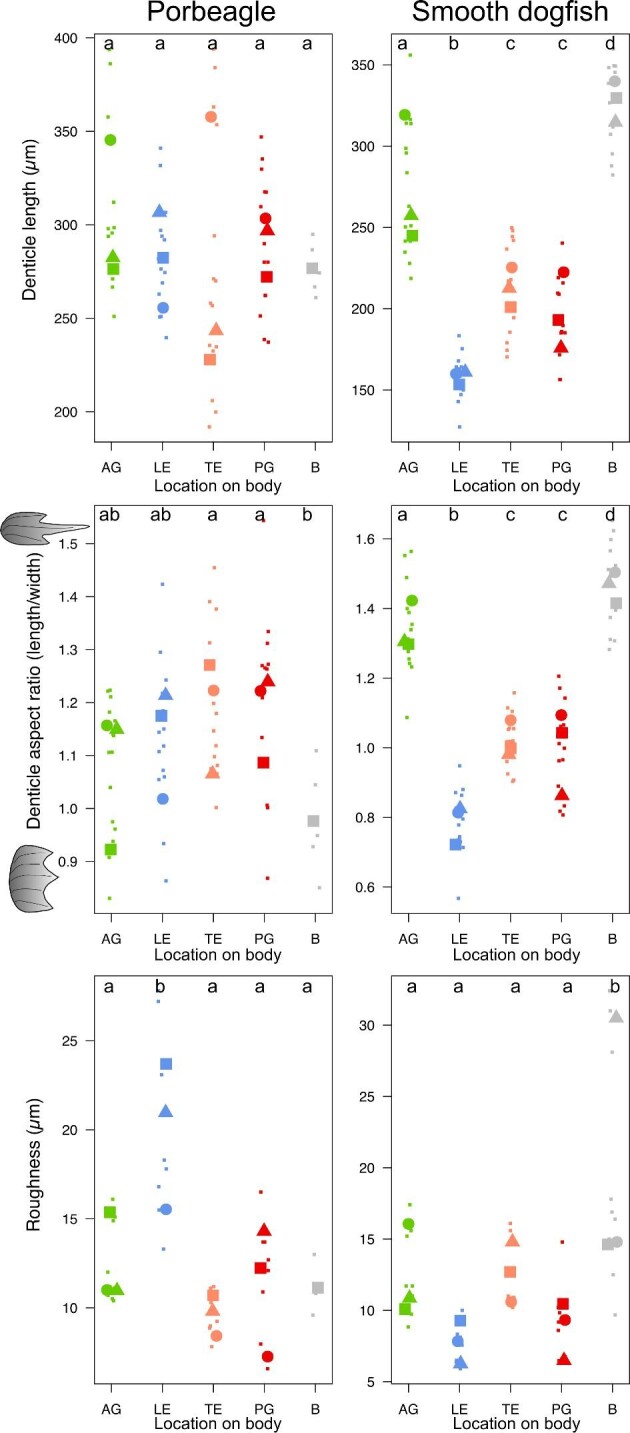
Denticle length, denticle aspect ratio, and roughness shown for porbeagle (*Lamna nasus*) and smooth dogfish (*Mustelus canis*) samples across all five sampled regions (see Fig. 1). Small points are each measurement and larger points are individual means with point shape indicating different individuals. The results of nested ANOVAs are provided at the top of each graph as significant groupings designated with different letters.

To better understand how denticle morphology varies across our five sampled regions, we compared measurements of morphology across regions in two representative species: the porbeagle shark and smooth dogfish (Fig. 4). In particular, we compared measurements of denticle length, denticle aspect ratio (length/width), and surface roughness (Fig. 4). We did not observe any significant differences in denticle length among all five regions in porbeagle sharks (nested ANOVA: F(4, 58) = 0.8972, *P* = 0.47); however, we found differences in denticle length among different regions in smooth dogfish (nested ANOVA: F(4, 68) = 115.50, *P* < 0.0001). In particular, we found that in smooth dogfish the B region (ventral to the dorsal fin, Fig. 1) had the longest denticles, followed by the AG region. Next in length were PG denticles, grouped together with the region at the TE of the interbranchial skin, and then the denticles at the LE of the interbranchial skin as the shortest in length (all indicated pairwise comparisons, *P* < 0.05).

Higher values of denticle aspect ratio indicate more elongate denticles, and we found significant differences among regions for both the porbeagle (nested ANOVA: F(4, 58) = 3.17, *P* = 0.02) and smooth dogfish (nested ANOVA: F(4, 68) = 111.52, *P* < 0.0001). The patterns in denticle aspect ratio were different in each species and the differences among regions were much weaker in the porbeagle (Fig. 4); in the porbeagle shark, B region denticles had a lower aspect ratio (stouter in shape) than the PG and TE regions (pairwise comparisons, *P* < 0.05), with AG and interbranchial skin LE regions intermediate and indistinguishable from all groups (pairwise comparisons, *P* > 0.05). In the smooth dogfish, B region denticles had the highest aspect ratio (are more elongate in shape) than the other four regions. The high aspect ratio of B region denticles is followed by the AG region, followed by a group containing both PG and interbranchial skin TE regions, and finally the interbranchial skin LE region with the lowest aspect ratio values (indicated pairwise differences *P* < 0.05).

Measurements of roughness for porbeagle and smooth dogfish surfaces also showed different patterns for the two species (Fig. 4). There were differences among regions for the porbeagle samples (nested ANOVA: F(4, 32) = 24.58, *P* < 0.0001) with the interbranchial skin LE region having a higher denticle surface roughness compared with the other four regions (indicated pairwise comparisons *P* < 0.05). There were also differences among regions for the smooth dogfish samples (nested ANOVA: F(4, 38) = 11.32, *P* < 0.0001), but in this species the B region had a higher roughness compared with the other four regions (indicated pairwise comparisons *P* < 0.05).

General differences in denticle morphology and surface topography are also observed among shark species examined and across all skin regions. Roughness values range from 3.0 μm at the PG region in the mako shark to 35.7 μm at the B region in the sand tiger (Table 1). Bonnethead denticles have relatively low roughness values across all regions (5.3–8.2 μm) compared with the high values observed in the sand tiger (28.4–35.7 μm; Table 1). In bonnethead, mako, porbeagle, smooth dogfish, and thresher sharks, skew values are negative, indicating textures that are dominated by valleys (Table 1). All skew values in the sand tiger shark are positive, meaning that these surfaces are more dominated by peaks than valleys. Sand tiger shark denticles are also the largest in size ranging from 306 μm (LE length) to 522 μm (B width), compared with the shortfin mako with the smallest denticles ranging from 131 μm (TE width) to 147 μm (TE length; Table 1).

**Table 1:** Comparative data on denticle surface morphology across the body from multiple shark species

Species	Location	Sq (μm)	Ssk	Sku	Sz (μm)	Length (μm)	Width (μm)	AR (μm)
Basking shark *(1)*	LE	24.0	1.60	6.0	146.0	441	293	1.5
*Cetorhinus maximus*	TE	32.5	1.09	3.7	170.7	466	283	1.6
Bonnethead shark *(1)*	AG	5.3	0.19	2.7	35.4	146	143	1.0
*Sphyrna tiburo*	LE	6.8	0.06	2.4	36.7	160	146	1.1
	TE	7.0	−0.02	2.7	42.0	167	173	1.0
	PG	8.2	0.05	2.8	48.0	164	174	0.9
	B	5.9	−0.08	2.6	34.3	201	186	1.1
Chain catshark *(3)*	AG	19.8	−0.06	2.5	119.4	515	368	1.4
*Scyliorhinus retifer*	LE	15.5	−0.22	2.9	120.0	281	255	1.1
	TE	17.2	−0.14	2.8	139.5	332	260	1.3
	PG	18.4	−0.15	2.5	116.0	477	310	1.5
	B	18.4	−0.06	2.7	133.0	559	333	1.7
Leopard shark *(1)*	AG	30.1	−0.46	2.7	179.3	366	278	1.3
*Triakis semifasciata*	LE	12.2	−0.44	3.9	108.8	204	200	1.0
	TE	16.2	−0.20	2.9	104.2	234	207	1.1
	PG	15.1	0.13	2.5	86.9	260	241	1.1
	B	15.5	0.24	2.9	118.7	463	327	1.4
Porbeagle *(3)*	AG	12.4	−0.14	2.8	81.1	301	282	1.1
*Lamna nasus*	LE	20.1	−0.18	2.6	120.5	282	249	1.1
	TE	9.6	−0.58	3.5	63.8	276	234	1.2
	PG	11.3	−0.43	3.4	86.6	291	248	1.2
	B	11.1	−0.21	2.8	63.1	277	285	1.0
Sand tiger *(1)*	AG	34.0	0.42	2.6	192.0	425	449	0.9
*Carcharhinus taurus*	LE	34.8	0.36	2.3	216.7	306	446	0.7
	TE	28.4	0.19	2.5	146.0	367	469	0.8
	PG	32.5	0.36	2.3	172.7	370	441	0.8
	B	35.7	0.38	2.1	149.0	518	522	1.0
Shortfin mako *(2)*	AG	6.8	−0.73	3.8	47.5	178	142	1.3
*Isurus oxyrinchus*	LE	10.3	−0.20	2.9	68.3	177	163	1.1
	TE	5.4	−0.61	3.9	38.9	147	131	1.1
	PG	3.0	−0.28	4.7	27.6	162	146	1.1
	B	10.9	−1.00	5.8	99.7	191	158	1.2
Silky shark *(1)*	LE	10.4	−0.25	2.8	67.2	205	251	0.8
*Carcharhinus falciformis*	TE	9.0	−0.16	2.8	61.3	177	200	0.9
Small-spotted catshark *(1)*	AG	20.2	−0.28	2.8	139.3	396	218	1.8
*Scyliorhinus canicula*	LE	11.6	−0.26	2.8	65.2	225	195	1.2
	TE	16.5	−0.55	3.8	143.7	315	203	1.6
Smooth dogfish *(3)*	AG	12.3	−0.28	2.7	76.2	274	204	1.3
*Mustelus canis*	LE	7.8	−0.50	3.1	50.0	158	202	0.8
	TE	12.7	−0.38	3.2	88.0	213	209	1.0
	PG	8.8	−0.22	3.4	71.1	197	198	1.0
	B	20.0	−0.77	3.7	131.4	328	225	1.5

**Table 1:** Continued.

Species	Location	Sq (μm)	Ssk	Sku	Sz (μm)	Length (μm)	Width (μm)	AR (μm)
Spiny dogfish *(1)*	AG	12.1	0.19	2.5	68.2	199	191	1.0
*Squalus acanthias*	LE	11.2	−0.11	2.4	77.5	188	212	0.9
	TE	12.8	−0.04	2.9	90.5	223	210	1.1
	PG	11.6	−0.37	3.4	89.0	208	176	1.2
	B	13.1	0.23	2.5	81.1	348	225	1.6
Thresher *(1)*	AG	7.4	−0.71	4.1	57.8	229	178	1.3
*Alopias vulpinus*	LE	18.2	−0.37	2.5	112.2	295	332	0.9
	TE	8.9	−0.38	2.8	55.9	173	136	1.3
	PG	10.9	−0.50	3.0	71.0	195	207	0.9
	B	7.1	−0.83	5.3	65.9	235	170	1.4
White shark *(1)*	AG	7.6	0.35	2.9	49.9	255	245	1.0
*Carcharodon carcharias*	LE	15.8	−0.15	2.5	94.8	248	252	1.0
	TE	7.3	−0.08	2.8	43.9	242	227	1.1
	PG	7.4	0.08	3.0	53.4	314	266	1.2
	B	10.7	−0.48	3.9	95.6	365	290	1.3

Numbers in parentheses indicate sample size.

Sq = roughness, Ssk = Skew, Sku = Kurtosis, Sz = maximum feature height, AR = aspect ratio (length/width).

AG = anterior to gill slit 1, LE = leading edge, TE = trailing edge, PG = posterior to gill slit 5, B = body (refer to Fig. 1).

### Differences in leading and trailing edge denticle morphology among species

**Fig. 5:**
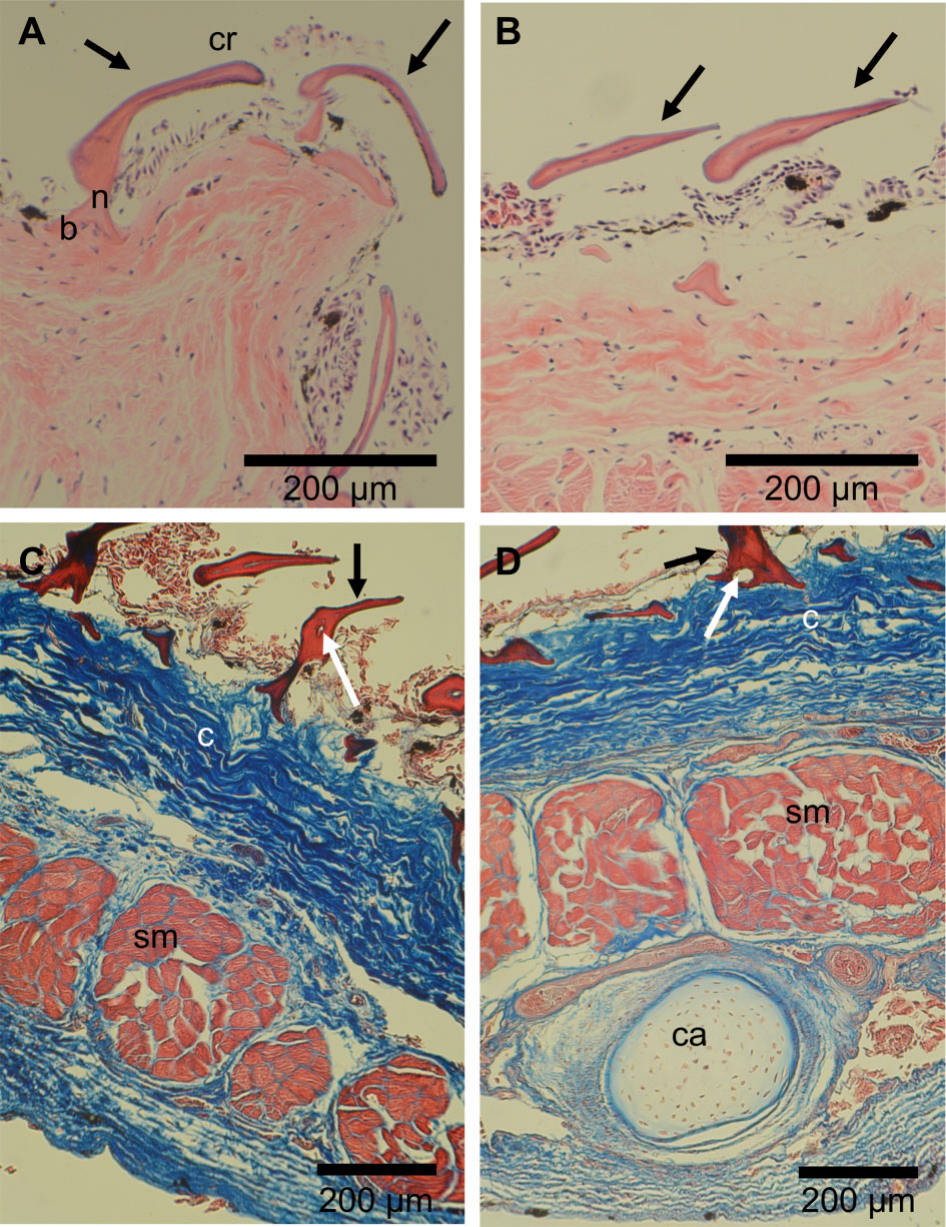
Denticle histology in *Scyliorhinus retifer*. All images are sagittal sections of the interbranchial skin region. **(A)** Leading edge denticles showing the crown (cr), neck (n), and base (b) of an individual denticle, H&E stain. **(B)** Trailing edge denticles, H&E stain. **(C)** Section of skin demonstrating the collagen fibers (c) of the dermis and the skeletal muscle bundles (sm) within the interbranchial pouch, Masson's trichrome. **(D)** Section of skin showing branchial supporting cartilage (ca) within the interbranchial skin region, Masson's trichrome. Black arrows (A–D) point to individual denticles; white arrows (C, D) point to the pulp cavity. Anterior is left and posterior is right.

The histological structure of interbranchial skin denticles was also investigated with the aim of comparing their anatomy to previously published descriptions of denticles from other body regions, and to establish the relationship of interbranchial denticles to underlying muscle and cartilage. Similar to dermal denticles found on the body, the interbranchial skin denticles also have a crown, neck and base embedded in the epithelium (Fig. 5). The smooth-edged LE denticles can be distinguished from the TE denticles based on the curvature at the crown (Fig. 5A and B). The interbranchial skin denticles also contain a pulp cavity (Fig. 5C and D). Histological sections of the interbranchial region demonstrate a thick layer of collagen fibers underlain by bundles of striated muscle and branchial cartilage (Fig. 5C and D).

**Fig. 6:**
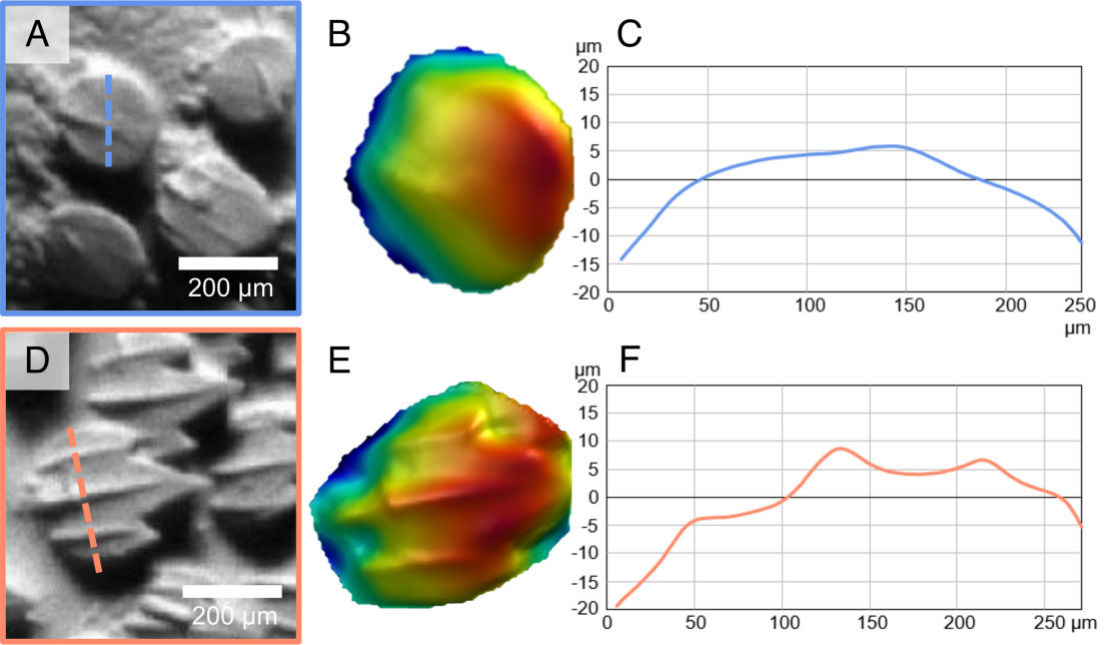
Three-dimensional surface profilometry images and profiles of two individual denticles from chain catshark (*Scyliorhinus retifer*) interbranchial skin. Panels **(A)** and **(D)** show an individual denticle from the leading edge (A) and trailing edge (D) of the interbranchial skin surface. Panels **(B)** and **(E)** show topography of each denticle's crown. Color shows the height of the surface, and both denticles are scaled to the same maximal height, with red representing the maximum height of 50 μm and blue the minimum height of 0 μm. Panels **(C)** and **(F)** show the height profiles across these two individual denticles (profile indicated by the dashed lines in A and D). Zero height is the mean surface height in these plots. The three surface ridges present on the trailing edge interbranchial denticle are clearly visible in panel F compared with the more subtle single ridge on the leading edge interbranchial denticle in panel C.

**Fig. 7:**
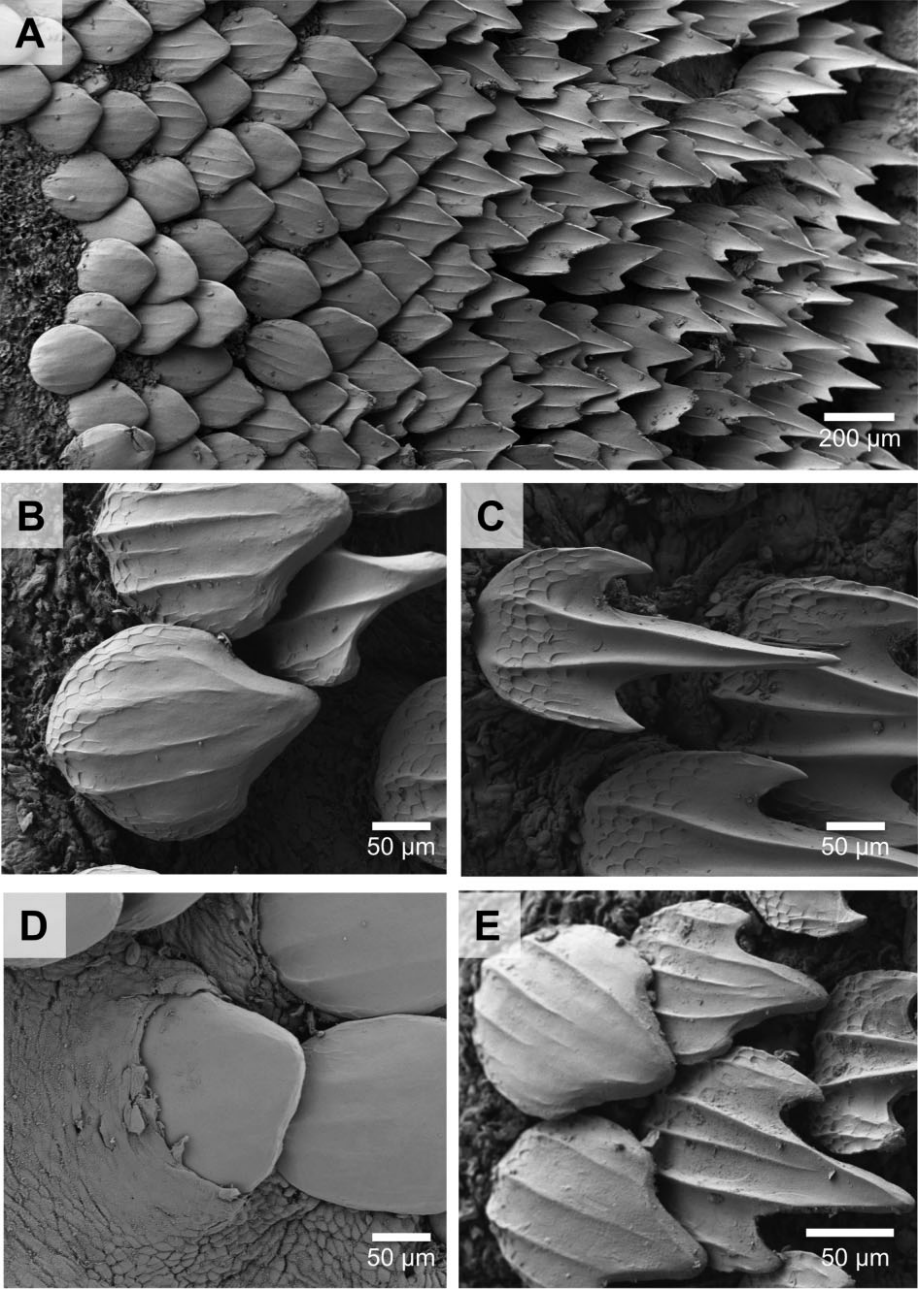
Scanning electron micrographs of denticles from the interbranchial skin of a chain catshark (*Scyliorhinus retifer*). Panel **(A)** shows the transition from the leading edge to the trailing edge denticle shapes on the interbranchial skin surface. Panels **(B)** and **(E)** demonstrate how rapidly (spatially) denticles change shape. Panel **(C)** shows a denticle at the trailing edge of the interbranchial skin, with surface ridges and tines (posterior spines). Panel **(D)** shows an erupting denticle at the leading edge, alongside other smooth-edged denticles.

Morphological differences between LE and TE denticle crown surfaces and profiles in the chain catshark are demonstrated using individual denticles (Fig. 6). The profile of the LE denticle exhibits fewer surface features than the TE denticle (Fig. 6C and F). The LE denticle has a small ridge in the center (Fig. 6A–C), while the TE denticle has three ridges (Fig. 6D–F). Further, the distal crown margin of the TE denticle has three posteriorly directed tines (i.e., prongs or sharp points), whereas the LE edge denticle is smooth-edged (Fig. 6). A dramatic gradient in denticle morphology and surface topography of the interbranchial skin is visualized in chain catsharks using scanning electron microscopy (Fig. 7A). Even over a short distance of 10 denticles or less, denticle shape changes from smooth-surfaced with rounded trailing edges to elongate with prominent posterior tines and surface ridges (Fig. 7). The LE denticles (Fig. 7B) on the interbranchial skin in the chain catshark are rounder and have less prominent ridges compared with the TE denticles (Fig. 7C). This transition in denticle morphology occurs even between adjacent denticles (Fig. 7E). We also observed the presence of denticles erupting through the epidermis on interbranchial skin (Fig. 7D).

**Fig. 8:**
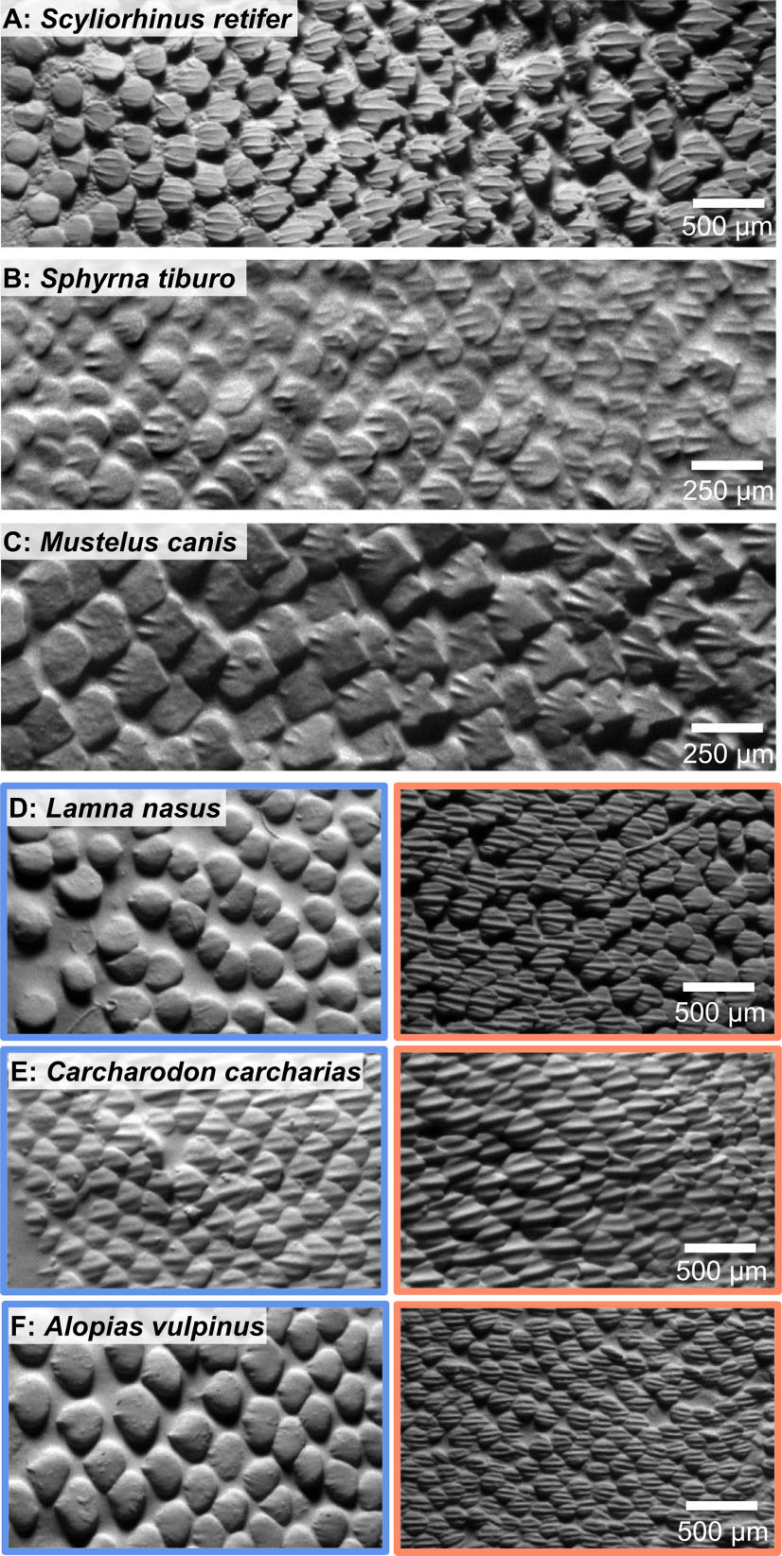
Grayscale images (from surface profilometry) of denticles on the interbranchial skin of six different shark species, illustrating the change in denticle morphology from the (left) leading edge to the (right) trailing edge. In panels **(A–C)**, the change in denticle morphology is apparent from the leading edge to the trailing edge in the small interbranchial region: anterior denticles have rounded margins in contrast to the pointed posterior margins of denticles on the trailing edge of the interbranchial skin. Panels **(D–F)** illustrate differences in denticle morphology from the leading edge (blue) to the trailing edge (orange) of the interbranchial skin in three additional species. The images had to be separated because the interbranchial distance in these large species is too great to be captured in one image. Flow in all images would be from left to right.

**Fig. 9:**
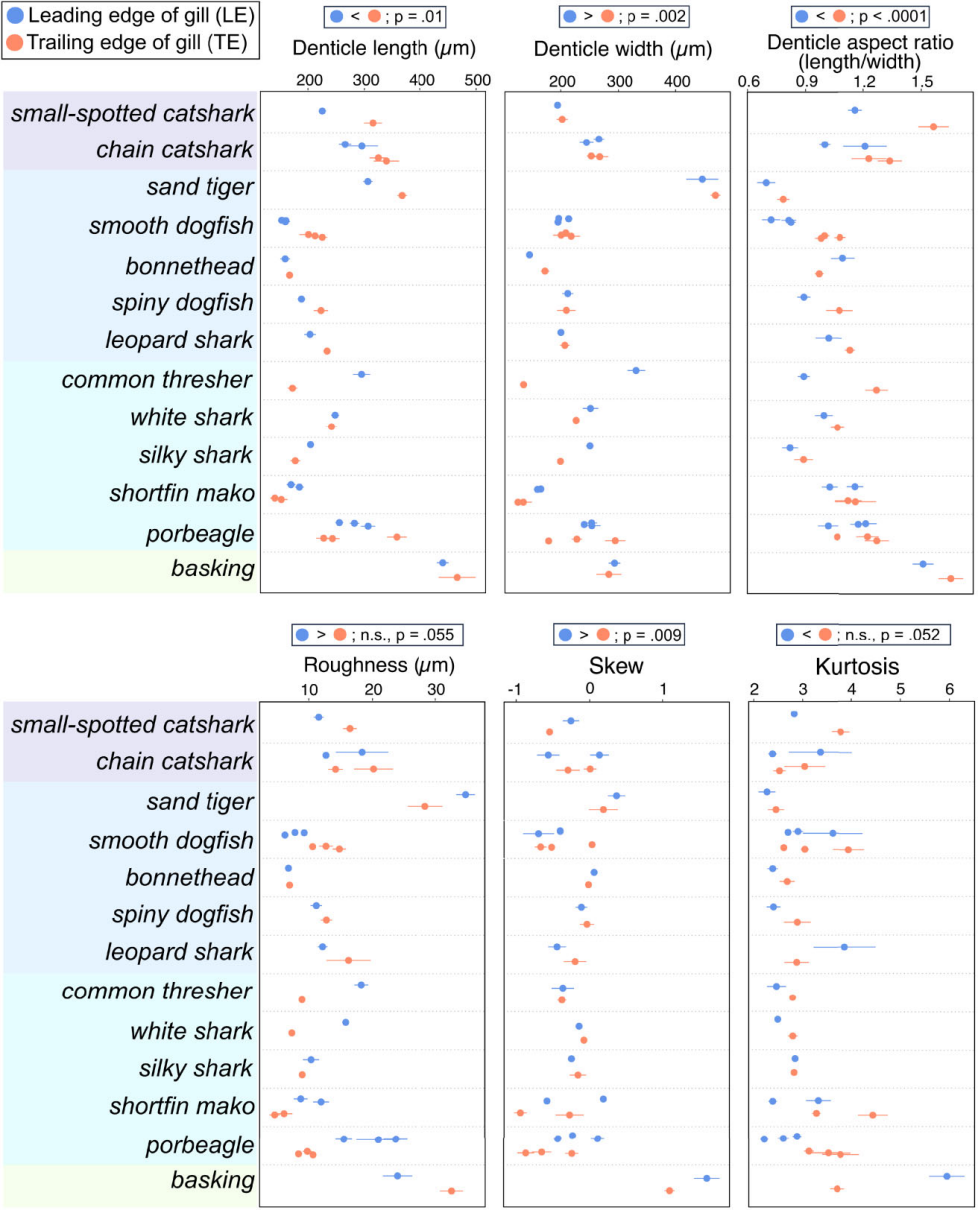
Individual means with error bars (+/− one standard error of the mean) plotted by species for denticle length, width, aspect ratio, roughness, skew and kurtosis (see Methods for measurement descriptions). Species names are shaded to represent four broad ecological categories: benthic (purple), demersal (blue), pelagic (teal) and suspension-feeding (green, from top to bottom). Results of nested ANOVAs where all species are pooled are provided above each graph. Additional nested ANOVAs where species are pooled by ecological category are presented in the Results.

The LE and TE denticles on the interbranchial skin differ qualitatively in morphology among all species of sharks studied (Fig. 8). For example, in most species (Fig. 8A, B, D, and F), LE denticles are rounder with few to no ridges. By pooling data across individuals and species for the LE and TE regions, we quantitatively investigated how denticle morphology and skin texture differed between these two regions (Fig. 9). We found significant differences in denticle morphology between LE and TE interbranchial skin regions, with the LE region having shorter denticles (denticle length nested ANOVA: F(1, 170) = 6.60, *P* = 0.0111), wider denticles (denticle width nested ANOVA: F(1, 170) = 9.39, *P* = 0.0025), and denticles with a lower aspect ratio (nested ANOVA: F(1, 170) = 48.97, *P* < 0.0001) compared with the TE region (Fig. 9; Table 1).

We also found significant differences in surface texture between the LE and TE interbranchial skin regions, with the LE having higher skew values (more dominated by peaks and other positive surface features) compared with the TE (nested ANOVA: F(1, 94) = 7.08, *P* = 0.0092; Fig. 9; Table 1). The patterns for roughness and kurtosis approached significance but were not significant at the 0.05 level (nested ANOVA for roughness: F(1, 94) = 3.77, *P* = 0.0551; nested ANOVA for kurtosis: F(1, 94) = 3.88, *P* = 0.0519). The general pattern for these two variables was that LE surfaces tended to have a higher roughness and lower kurtosis (less extreme values) compared with the interbranchial skin TE surfaces.

When grouping LE and TE data by ecological categories (e.g., benthic LE vs. benthic TE vs. demersal LE, etc.), there were statistical differences in denticle morphology and surface texture between the LE and TE regions in different ecologies (nested ANOVA for denticle length: F(7, 164) = 11.98, *P* < 0.001; Fig. 9). Pairwise post-hoc comparisons show that demersal and benthic species have shorter LE denticles compared with TE, whereas pelagic species have longer LE denticles than TE denticles. Suspension feeders have LE and TE denticles of similar lengths. Pelagic species also have LE denticles that are wider than their TE denticles, while all other ecological groupings have LE that are similar in width to the TE denticles (nested ANOVA for denticle width: F(7, 164) = 6.64, *P* < 0.0001). Aspect ratio in all groups except for suspension feeders is lower in LE denticles than TE (LE denticles are less elongate in the anteroposterior direction); suspension feeders have aspect ratios similar between LE and TE denticles (nested ANOVA for aspect ratio: F(7, 164) = 12.14, *P* < 0.0001). Roughness and skew values showed similar trends with demersal and benthic species having equal LE and TE denticle values, pelagic LE denticles having higher values compared with TE, and LE denticles having lower values than the TE denticles in suspension feeders (nested ANOVA for roughness: F(7, 88) = 13.13, *P* < 0.0001 and nested ANOVA for skew: F(7, 88) = 7.89, *P* < 0.0001). Kurtosis values also differed among the ecological groups (nested ANOVA for kurtosis: F(7, 88) = 10.00, *P* < 0.0001), with pelagic species having lower kurtosis at the LE, suspension feeders having higher kurtosis at the LE, and benthic and demersal species having indistinguishable kurtosis at LE versus TE regions.

**Fig. 10:**
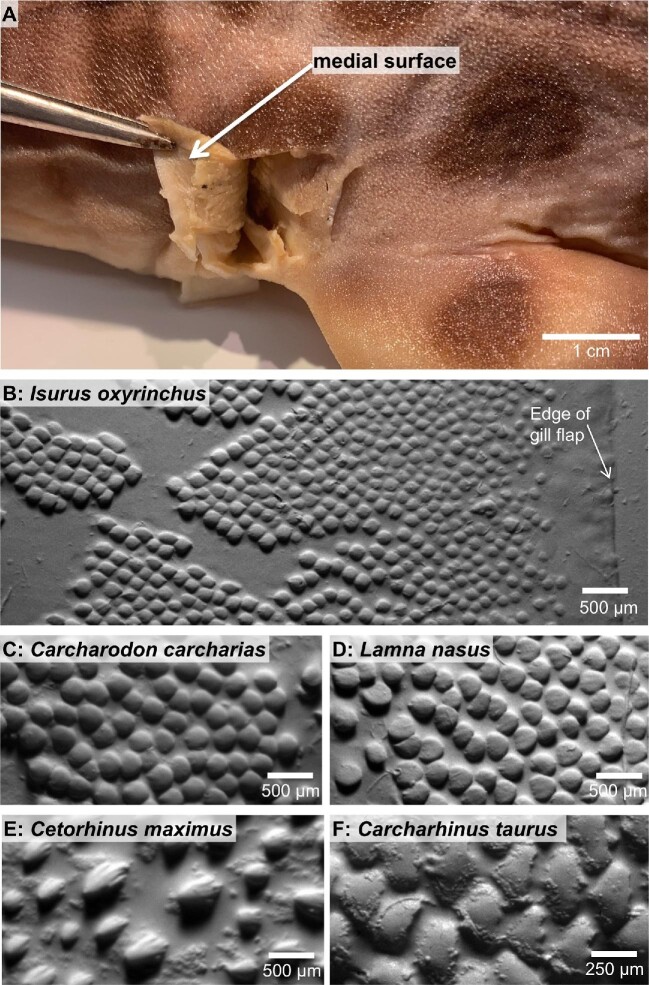
Images demonstrating the presence of denticles on the inner (medial) surface of interbranchial skin. **(A)** Photograph of a leopard shark (*Triakis semifasciata*) demonstrating the medial surface of the interbranchial skin region. The tissue between gill slits 3 and 4 has been cut and is being folded toward the head with forceps to reveal the medial surface. **(B–F)** Grayscale images of the denticles on the medial surface of the interbranchial skin for five shark species (from surface profilometry). Left is posterior and right is anterior.

Denticles were also observed on the medial surface of the interbranchial skin (Fig. 10). In all species of sharks studied, denticles were present on the posterior trailing edges of the medial gill flap surface. Though morphological and surface metrology measurements were not collected, these denticles resemble the LE denticles on the lateral surface of the interbranchial skin as they are smooth-edged and lack ridges on the crowns (Fig. 10B–F).

## Discussion

This is the first comparative study of denticle morphology and surface topography on the interbranchial skin in a wide diversity of shark species. We show that the interbranchial skin region exhibits a considerable transition in denticle shape and ornamentation over just a short distance, and that denticle variation in this one small region is the equivalent of that seen around the body as a whole (Ankhelyi et al. 2018; Reif 1985b). Our statistical results show that when leading and trailing edge (LE and TE) sites are pooled across species, we see significant differences in denticle length, width, aspect ratio, and surface skew, along with results that are approaching significance in surface roughness and kurtosis. In particular, LE denticles tend to be shorter in length, broader in width, less elongate, and have higher skew (tend to have more peaks on their surface) compared with TE denticles from the interbranchial skin region. An additional novel result from the current study was the discovery of smooth-edged and ridge-less denticles on the inner (medial) surface of interbranchial skin patches (Fig. 10).

### Morphological diversity of denticles on the interbranchial skin in shark species

While the diversity of shark denticles on the body is well described in the literature (e.g., Ankhelyi et al. 2018; Bigelow and Schroeder 1948; Díez et al. 2015; Lang et al. 2012; Motta et al. 2012; Oeffner and Lauder 2012; Raschi and Tabit 1992; Reif 1985b), as researchers continue to explore shark skin surfaces, some surprising features of denticle diversity have emerged. Denticle morphology has been shown to differ across shark bodies, with quantifiable and repeated differences in denticle form between leading edge and trailing edge locations on the body and fins of multiple species (Ankhelyi et al. 2018; Motta et al. 2012; Popp et al. 2020; Raschi and Tabit 1992; Reif 1985b). Additionally, the recent addition of 3D imaging methods (e.g., gel-based profilometry, micro computed-tomography) has provided a richer understanding of quantitative denticle morphology and surface diversity across species and body locations (Ankhelyi et al. 2018; Domel et al. 2018; Popp et al. 2020; Wainwright et al. 2017). New discoveries continue to be made about shark denticle diversity and form; recently, Tomita et al. (2020) described denticles on the eye surface of whale sharks, and presumably these denticles function in abrasion resistance and protection of the eye. Additionally, a single previous image demonstrated a surprising transition in denticle shape on the interbranchial skin of one shark (smooth dogfish, *Mustelus canis*; Ankhelyi et al. 2018). In this image, changes in denticle crown shape that normally occur across distinct body regions were observed within only a few millimeters—an observation that inspired this study.

When comparing LE and TE interbranchial skin denticles, all species display a morphological transition from denticles with a spatulate shape, rounded distal margins, and either reduced or no ridges, to more elongate, ridged denticles with posterior tines. In some species, such as the chain catshark (Fig. 7), this transition is particularly dramatic and denticles within a few hundred microns can display substantially different morphology. When data were pooled between LE and TE sites across species, LE denticles have shorter and broader crown lengths and higher skew values compared with TE denticles from the interbranchial skin region. Although we find significant trends when pooling data from all species, in many cases these variables show mixed trends when looking across individual species (Figs. 4 and 9); we discuss these different trends with respect to general ecological categories.

Pooling LE and TE data separately into ecological groups provides a more refined perspective on how ecology and ventilation mode may affect denticle morphology at the interbranchial region (Fig. 9). We show that across ecologies, denticle shape (aspect ratio) shows consistent differences between LE and TE sites, with nearly all groups showing stouter, less-elongate denticles at the LE compared with TE. However, patterns of denticle size (length and width) are different among ecologies; for example, ram-ventilating pelagic sharks have larger LE denticles compared with TE denticles, whereas the opposite is true in active-ventilating benthic and demersal sharks. The larger LE denticles of the pelagic group may also contribute to the significantly higher roughness values at the LE versus the TE in this ecological group, whereas benthic and demersal ecologies have no significant differences in LE versus TE roughness. These differences combined with different patterns across ecologies in skew and kurtosis demonstrate that although denticle shape shows a consistent pattern between LE and TE sites across ecological groups, measurements of denticle size and surface form indicate that the ram-ventilating pelagic sharks have different patterns in LE vs. TE morphology compared with active-ventilating ecological groups. These patterns strongly suggest that ventilatory ecology plays a role in shaping the morphology and function of denticles on the interbranchial skin, but that denticle shape may function in a way that is consistent across ecology or ventilatory mode.

Our single suspension-feeding species (basking shark) also shows different trends among TE and LE morphology compared with other ecological groups, and this species is notably different from pelagic sharks, despite inhabiting the same general environment. These differences in results suggest that filter feeding, as well as the slow swimming speed of these species, influences the morphology and possible function of interbranchial denticles compared with other ram ventilators (e.g., thresher and white shark; Cheer et al. 2001).

Previous studies that have measured shark skin using GelSight reported largely negative skew values, indicating that these surface textures are more dominated by valleys or pits than peak-like surface features (Ankhelyi et al. 2018; Popp et al. 2020). Interestingly, interbranchial skin denticles from the basking shark and sand tiger have positive skew values (ranging from 0.19 to 1.6: Table 1), suggesting that these surfaces are dominated by positive surface features (i.e., peaks or features above the mean height). Other surfaces with similar positive skew values are the skin surfaces of bony fishes (Wainwright et al. 2017).

### Comparing leading and trailing edge denticles beyond the interbranchial region

Previous studies (Ankhelyi et al. 2018; Motta et al. 2012; Popp et al. 2020; Reif 1985b) have noticed that there are often repeated patterns in denticle morphology and surface texture when comparing leading and trailing edge sites on the body of sharks (nose vs. tail) or on individual fins (leading vs. trailing edges). Here, we discuss how these patterns compare with the trends seen here on the interbranchial skin.

When our LE and TE data are separately pooled across all species, LE denticles tend to have higher roughness values compared with TE denticles (Fig. 9 and Table 1). Previous literature demonstrates similar trends on leading and trailing edges of different body parts; many shark species (smooth dogfish, leopard shark, gulper shark, thresher shark) have higher roughness values on leading edge surfaces compared with trailing edge surfaces across various sites (nose, tail, tail tip, dorsal fin, and pectoral fin; Ankhelyi et al. 2018; Popp et al. 2020). In addition, most leading edge sites in other species have much larger denticles compared with their relevant trailing edges (Ankhelyi et al. 2018; Popp et al. 2020)—we see this pattern repeated in a subset of our species here, specifically those species with a pelagic ecology. We also note that leading edge sites both on interbranchial skin and across other body regions tend to have denticles with reduced surface ridges and more rounded posterior edges (Ankhelyi et al. 2018; Popp et al. 2020).

These repeated patterns across the body, fins, and at the interbranchial skin suggest that both leading and trailing edges may share similar functional pressures across different body parts and species. Perhaps free-stream flow over the body and fins and flow passing over interbranchial skin imposes similar hydrodynamic constraints on denticle shape and surface texture. In addition, it has been postulated that the unique shape of the leading edge denticles on shark fins and tails could also provide protection to reduce damage at leading edge sites (Ankhelyi et al. 2018; Popp et al. 2020).

### Functional significance of interbranchial skin denticles

The consistent differences observed between leading and trailing edge interbranchial skin denticles on a diversity of shark species suggests two non-mutually exclusive hypotheses for how these interbranchial skin denticles might function. First, the smooth-edged crowns and lack of ridges on leading edge interbranchial skin denticles may act to reduce friction from contact with the preceding gill flap. During respiration, motion of the gill flaps results in contact between neighboring interbranchial regions, particularly when gill slits are closed when buccal expansion moves water through the mouth and into the buccal cavity. In benthic sharks such as the chain catshark, active respiratory pumping involves not just a buccal pump, but also activity in muscles located within the interbranchial skin (Fig. 6) to constrict the gill pouches and force water posteriorly out of the gill slits during the branchial expansive phase of respiration (Brainerd and Ferry-Graham 2006; Ferry-Graham 1999). This active motion results in repeated contact of the posterior interbranchial margin with anterior denticles on the downstream interbranchial-skin segment. Reduction of ridges and the presence of smooth posterior margins on these leading edge interbranchial skin denticles could reduce friction and damage to gill flaps in the region where physical contact occurs with regularity during ram ventilation. At interbranchial regions such as the TE (Fig. 1), where no physical contact occurs during respiration, denticles have a more classic shape with prominent ridges and posteriorly directed pointed tines (e.g., Fig. 8).

A second hypothesis about why denticles transition in morphology at the interbranchial skin region focuses on the possibility that the transition in denticle shapes on the interbranchial surface serves to reduce fluid dynamic drag resulting from respiration. Respiratory flows in species with either pulsatile or ram ventilation (Roberts 1975; Wegner et al. 2012), likely result in interbranchial skin denticles being subjected to complex flow patterns over their surface that necessarily create drag. Drag forces could be reduced by altering flow close to the surface both near and within the boundary layer. For example, the transition from smooth to ridged denticles as flow exits the gill slits could help maintain a laminar flow condition and reduce friction. Alternatively, the transition could create turbulence to instead prevent flow separation (Smits 2000), reducing drag forces on the posterior margins of the gill flaps that can undergo significant movement during respiration. Hydrodynamic drag has been suggested to decrease with the presence of ridges on manufactured riblets (Bechert et al. 2000; Bechert and Hage 2007). Although water flow patterns near the interbranchial skin have yet to be studied experimentally, the rapid transition in denticle shape suggests that this area may be a fruitful location to investigate the relationship between flow and denticle shape.

To date, analyses of the relationship between denticle shape and water flow patterns in sharks have necessarily been inferential, as it has not been possible to study water movement over shark denticles *in vivo* in freely swimming sharks at the resolution needed to visualize flow over individual denticles. The current study demonstrates that there are gradients in denticle morphology and surface topography on the interbranchial skin in a variety of shark species. These results, along with previous studies demonstrating the potential hydrodynamic function of dermal denticles, provide the foundation to further investigate the relationship between denticle morphology and water flow. To test hypotheses concerning the hydrodynamic effects of denticle diversity, the interbranchial gill skin of benthic sharks, like the chain catshark, may prove to be a valuable experimental model. Benthic sharks are active ventilators and will pump water over their gills when they are not swimming. This behavior combined with their sedentary nature may provide conditions where *in vivo* ventilatory flows in benthic sharks can be imaged and measured, even over the small (2–4 mm) segments of the interbranchial skin. Our results also show that benthic sharks, such as the chain catshark (Fig. 7), exhibit dramatic gradients in denticle shape on interbranchial skin that are similar to patterns in other species, making benthic sharks potentially representative as a model in this context.

Visualization of respiratory flows exiting gill slits in laboratory experiments could help to better understand how denticle shape affects water flow patterns. For example, if the flow at the leading edge of an interbranchial segment is different than the flow at the trailing edge, this provides some support for our hypothesis that the change in denticle shape between these regions may be due to hydrodynamic effects. We can make predictions, such as—denticles that experience more complex turbulent flows may have increased surface ornamentation (ridges and posterior tines), which we can then test by correlating aspects of surface flow with the patterns in denticle diversity we have shown in this manuscript. Furthermore, advances in additive manufacturing may make it possible to use our morphological data to create models of denticles with different morphology that can then be systematically tested in different flow conditions, either physically or through computational means. These types of studies may be able to more directly connect denticle diversity and drag reduction in different flows. We hope our demonstration of denticle diversity at the interbranchial region of sharks inspires future work on the relationships between form and function in shark skin denticles.

## Supplementary Material

obab034_Supplemental_File

## Data Availability

The data that support the findings of this study are available from the corresponding authors upon request.
