# Supplementary material for: Dermal Denticle Diversity in Sharks: Novel Patterns on the Interbranchial Skin
Source: Integr Org Biol. 2021 Dec 22;3(1):obab034. doi: 10.1093/iob/obab034 (PMC8694198; doi:10.1093/iob/obab034)
Supplement: obab034_Supplemental_File [file obab034_supplemental_file.pdf]

Supplemental Table 1. Skin samples from each individual shark used for denticle morphology and surface topography quantification.

Species	Anterior to gill 1 (AG)	Interbranchial skin (LE, TE)	Posterior to gill 5 (PG)	Body (B)
Alopias vulpinus				
Thresher shark	✔	✔	✔	✔
Carcharhinus falciformes				
Silky shark		✔		
Carcharhinus taurus				
Sand tiger shark	✔	✔	✔	✔
Carcharodon carcharias				
White shark	✔	✔	✔	✔
Cetorhinus maximus				
Basking shark		✔		
Isurus oxyrinchus				
Mako shark 1	✔	✔	✔	
Mako shark 2	✔	✔	✔	✔
Lamna nasus				
Porbeagle 1	✔	✔	✔	✔
Porbeagle 2	✔	✔	✔	
Porbeagle 3	✔	✔	✔	
Mustelus canis				
Smooth dogfish 1	✔	✔	✔	✔
Smooth dogfish 2	✔	✔	✔	✔
Smooth dogfish 3	✔	✔	✔	✔
Scyliorhinus canicula				
Small spotted catshark	✔	✔	✔	✔
Scyliorhinus retifer				
Chain catshark 1	✔	✔	✔	✔
Chain catshark 2	✔	✔	✔	✔
Chain catshark 3				✔
Sphyrna tiburo				
Bonnethead	✔	✔	✔	✔
Squalus acanthias				
Spiny dogfish	✔	✔	✔	✔
Triakis semifasciata				
Leopard shark	✔	✔	✔	✔
